# Pathology and regulation for research in the UK: an overview

**DOI:** 10.12688/f1000research.19732.2

**Published:** 2019-09-19

**Authors:** Owen John Driskell, Jessica L. Lee, Karin A. Oien, Andy Hall, Clare Verrill, CM-Path QA Panel

**Affiliations:** 1 Department of Clinical Biochemistry, University Hospitals of North Midlands, Stoke-on-Trent,, Staffordshire, UK; 2 Institute for Applied Clinical Sciences, University of Keele, Stoke-on-Trent, Staffordshire, UK; 3 National Institute for Health Research Clinical Research Network West Midlands, NIHR CRN, Albrighton, Shropshire, UK; 4 Strategy and Initiatives, National Cancer Research Institute, London, UK; 5 Institute of Cancer Sciences, University of Glasgow, Glasgow, UK; 6 Newcastle University, Newcastle, UK; 7 Nuffield Department of Surgical Sciences, University of Oxford, Oxford, UK

**Keywords:** Research regulation, Research governance, MHRA, GCP, HTA, Quality Management, Clinical Trial, CTIMP

## Abstract

The input of pathologists is essential for the conduct of many forms of research, including clinical trials. As the custodians of patient samples, pathology departments have a duty to ensure compliance with the relevant regulations, standards and guidelines to ensure the ethical and effective use for their intended investigational analysis, including when patients are participating in a research study. The results of research studies have impacts beyond the research study itself as they may inform changes in policy and practice or support the licensing of medicines and devices. Compliance with regulations and standards provides public assurance that the rights, safety and wellbeing of research participants are protected, that the data have been collected and processed to ensure their integrity and that the research will achieve its purpose. The requirements of the regulatory environment should not be seen as a barrier to research and should not significantly impact on the work of the laboratory once established and integrated into practice. This paper highlights important regulations, policy, standards and available guidance documents that apply to research involving NHS pathology departments and academic laboratories that are contributing to research involving human subjects.

## Introduction

**Figure 1.**
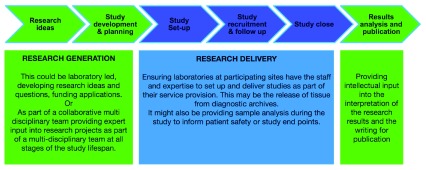
Involvement of pathology along the research study pathway. The pathology laboratory has different potential functions along the study pathway. Pathologists may be leading the Research Generation (green), e.g. coming up with the research question, applying for funding, writing up the research or providing pathology expertise to these activities as part of a multidisciplinary team. Pathologists may also be responsible for providing supporting Research Delivery activities (blue) for studies from outside researchers, e.g. assessing research protocols for local delivery and providing service activities such as reporting on or arranging the release of diagnostic tissue to research centres.

Evidence from research is needed to justify changes in practice and is a driving force behind improvements in patient care. Pathology can be involved throughout the research pathway - from developing research ideas, designing, setting up and running research studies, to analysing samples, interpreting the data and publishing the results ( Figure 1).

In addition to research initiated by pathologists, a large amount of research is led by researchers from outside pathology requiring services delivered by pathology departments. This research often requires services delivered by pathology departments. For some pathology staff this service delivery, for example the release of material from hospital archives, may be their only experience of research. Data generated from pathology departments support research in a wide variety of ways. This could be informing study eligibility assessments, companion diagnostics, pharmacokinetics, surrogate or other endpoints or the provision of data, results or tissue for further assessment. Results may already be in the health record as part of routine care or they may require further analysis or the release of tissue to central laboratories for a standardised assessment.

As well as the increase in the overall workload of pathology departments, there have been increases in clinical trials requiring pathology input. As research needs to be carried out in accordance with the appropriate regulatory requirements and standards, pathology departments need to have risk-based and pragmatic procedures in place to manage these requests. Regulations change and so it is important that pathology departments maintain an understanding of the current regulatory environment around research.

## UK Policy Framework for Health and Social Care Research

In the NHS research should be carried out in accordance with the UK Policy Framework for Health and Social Care Research ^1^, which replaced the Department of Health Research Governance Framework in October 2017. The Framework sets out the principles of best practice for the management and conduct of all health and social care research in the UK, to help organisations meet their legal requirements. It covers research in the NHS and other health and social care environments across all four nations. The Framework includes fifteen principles that apply to all research and a further four that apply only to interventional research. It provides a clear definition and outline of the roles and responsibilities for individuals and organisations involved in health and social care research including the sponsor, the funder, the research team, research sites, clinical research organisations and regulators. It states there should be clear designation of responsibility and accountability with clear lines of communication between all those involved in research.

The Framework is not a regulation - it is statutory guidance published in order to help organisations meet their legal obligations. It is a common set of principles that apply to any study. Though they aren’t identical, these will be recognisable to those already familiar with the principles of Good Clinical Practice. The framework is an important document for all those involved in NHS research as it provides a common language for research and enhances the possibilities for a unified and streamlined approach to research delivery.

## Good Clinical Practice, the UK Clinical Trials Regulations and the MHRA

The International Council for Harmonisation (ICH) of Technical Requirements for Pharmaceuticals for Human Use Good Clinical Practice (GCP) is an international standard for the design, conduct, performance, monitoring, auditing, recording, analysis, and reporting of clinical trials. These internationally recognised standards followed on from the Nuremberg Code and originate from the World Medical Association Declaration of Helsinki 1964 and were an attempt to overcome international inconsistencies in GCP ^2^.

The Guideline for Good Clinical Practice E6 from the European Medicines Agency includes the 13 Principles of ICH GCP ^3^. These guidelines refer to ‘clinical trials’ but may also be applied to other clinical investigations that may have an impact on the safety and well-being of human participants.

ICH GCP was incorporated into EU Directives 2001/20/EC and 2005/28/EC aimed at harmonising the regulation of Clinical Trials in the EU ^4, 5^. These were transposed into UK Law via The Medicines for Human Use (Clinical Trials) Regulations in 2004 ^6^ revised in 2006 ^7^ and have since been further amended. The UK has adopted the principles of GCP, but an additional principle relating to sponsor indemnity was added. This did not define ICH GCP as the legal standard unlike other European countries. As with the UK Policy Framework for Health and Social Care Research these regulations also include definitions and roles and responsibilities for research.

The Clinical Trial regulations define a clinical trial of an investigational medicinal product (CTIMP) as any investigation in human subjects, other than a non-interventional trial, intended (a) to discover or verify the clinical, pharmacological or other pharmacodynamic effects of one or more medicinal products, (b) to identify any adverse reactions to one or more such products, or (c) to study absorption, distribution, metabolism and excretion of one or more such products, with the object of ascertaining the safety or efficacy of those products. The Medicines and Healthcare products Regulatory Agency (MHRA) provide a tool for whether research is a CTIMP ^8^ and can provide further assistance if required.

The regulations have had subsequent amendments in response to developments in healthcare, for example research interventions in pandemic disease and advanced therapies such as gene therapy and tissue engineering. Europe will be implementing a new Clinical Trial Regulation (536/2014) ^9^ during 2020 and this may be implemented in the UK dependent on Brexit negotiations and future agreements. If the new Regulation does not come into force during the implementation period, the Government has confirmed that UK law will remain aligned with parts of the EU’s CTR legislation that are within the UK’s control, in order that researchers conducting clinical trials can plan with greater certainty ^10^.

The Medicines for Human Use Regulations permit inspection of clinical trials and their conduct, including work undertaken in a laboratory, by the MHRA GCP Inspectorate. It is therefore prudent for a laboratory to know which trials they support are CTIMPs and so are subject to these regulations and potential inspection.

The primary reference for describing the requirements of GCP in the context of laboratories is the EMA Reflection Paper for Laboratories that Perform the Analysis or Evaluation of Clinical Trial Samples published in 2012 ^11^. This was developed based on the MHRA Guidance on the Maintenance of Regulatory Compliance in Laboratories that Perform the Analysis or Evaluation of Clinical Trial Samples (published in 2009 now withdrawn). This document describes expectations for laboratories involved in the analysis of samples originating from a CTIMP.

The primary focus of inspection by the MHRA GCP Inspectorate is the analysis of samples that support primary and secondary endpoints and objectives of the trial. Although routine sample analyses for safety testing within clinical chemistry and haemotology laboratories are not the central focus of this inspection programme, the legislation does not differentiate between the purpose of different types of sample analysis; this is a pragmatic approach by the MHRA Inspectorate and potentially any aspects may be reviewed during an inspection. It is expected that laboratories consider and implement the guidance to support all forms of sample analysis linked to clinical trials whilst ensuring that this is appropriate and proportionate for the work being undertaken.

Further information in relation to the conduct of clinical trials can be found in the Good Clinical Practice Guide (otherwise known as the “Grey Guide”) produced by the MHRA ^12^. This covers all aspects of GCP and is a recommended reference for laboratories involved in clinical research (including those not involved in primary and secondary endpoint analysis). The MHRA also continues to publish blogs on a variety of topics including those directly relevant to laboratory involvement in CTIMPs ^13^.

## Human Tissue Act 2004 and the Human Tissue Authority

The Human Tissue Act (2004) relates to the removal, storage and use of ‘relevant material’ for Scheduled Purposes ^14^. The Act makes consent the fundamental principle to the use of human tissue. Although there are caveats, relevant material generally refers to samples, other than gametes, which include intact human cells. This includes most types of samples held in histology departments. Samples rendered acellular are not considered to be relevant material. There are eleven Scheduled Purposes for which consent is required, including ‘Research in connection with disorders, or the functioning, of the human body’. Samples collected as part of routine clinical care are not covered by the Human Tissue Act though they could subsequently be used for a Scheduled Purpose. The Act only applies in England, Wales and Northern Ireland as Scotland has separate legislation which only applies to samples collected from the deceased ^15^.

The Human Tissue Authority (HTA) was created by the Human Tissue Act to regulate the removal, storage and use of human tissue. They grant licences to establishments for a range of specific activities involving bodies and relevant material, including undertaking post-mortem examinations and storage of relevant material for Scheduled Purposes. HTA-licensed establishments include hospitals (both NHS and private), tissue banks, NHS blood and transfusion premises, private pathology services, clinical research facilities, commercial life sciences laboratories and universities.

Institutions handling human samples must ensure that they have any necessary licences for the activities carried out. The HTA does not license the ‘use’ of tissue for research or approve individual research projects or clinical trials. Neither do they have a role in the ethical approval of research. A research sector licence covers the storage of the relevant material. Storage of relevant material for research may be exempt from the need for a HTA licence if the sample is being stored according to the protocol of a research study approved by an NHS Research Ethics Committee. The HTA standards and guidance for research are contained within Code A: Guiding Principles and the fundamental principle of consent ^16^ and Code E: Research ^17^. There is a useful decision chart for whether you need a research licence on the HTA website (Code E Annex C).

## Other regulation

The regulations and guidelines that relate to research do not replace the existing regulations and guidelines that govern laboratory practice. All the existing professional standards and regulations still apply and those that relate specifically to research must be applied in addition to these. Examples include ISO-15189 ^18^, the Data Protection Act ^19^ implementing the General Data Protection Directive (GDPR) (as well as being custodian of patient samples Pathology is also custodian of their data) and the Transport of Dangerous Goods regulations ^20^. Researchers must be aware of, and adhere to, local NHS Trust policies and relevant professional standards such as those set by the General Medical Council (GMC), Royal College of Pathologists, the Institute of Biomedical Science (IBMS) and the Association of Clinical Scientists (ACS). These professional standards include the need to be appropriately qualified for their job roles and the need to maintain continued professional development mirroring requirements in research regulation. Further, international trials may be subject to the scrutiny of other agencies such as the US Food and Drug Administration (FDA) so it is important to consider differing regulatory requirements depending on the intended use of the data.

## Accreditation and research

NHS Pathology laboratories and those laboratories who are providing clinical services are used to what was Clinical Pathology Accreditation and is now Accreditation to ISO 15189 (Medical Laboratories - requirements for quality and competence in medical laboratories) ^18^ through the United Kingdom Accreditation Service (UKAS). These standards are about Quality Management supporting a culture and systems that can also be used to support the needs of research regulations and standards.

There are no laboratory accreditation schemes for the analysis of clinical trial samples run by the MHRA. For CTIMPs, laboratories are required to be compliant with all relevant legislation which includes working to the principles of GCP. Where analysis is undertaken for the purposes of patient safety during a CTIMP and is part of a routine repertoire of practice, UKAS accreditation is taken into account. The regulations permit this work to be inspected but often there are other activities which are prioritised for inspection due to the risk-based approach taken. Study sponsors frequently ask for the UKAS accreditation status of a clinical laboratory as part of their study set up process. However, ISO 15189 itself makes no specific mention of research. While the standards are complementary, laboratory practice also needs to quality manage areas of research practice not covered by ISO 15189. Examples include provision for the management of result reporting in CTIMP studies where blinding may be compromised, data integrity considerations ^21^ and the strict processing of trial samples according to the trial protocol.

Laboratories can be accredited to Good Clinical Laboratory Practice (GCLP) by third party providers. GCLP is a term for various guidance documents with their origins in a set of standards produced by the World Health Organisation (WHO) for the purposes of supporting safety and efficacy in international studies in developing countries ^22^ and subsequently reproduced by a number of non-government organisations ^23^. Good Laboratory Practice (GLP) applies to all non-clinical safety studies which are designed to determine the effects of a chemical on human health, animal health and the environment. GLP specifically excludes clinical experiments. Both GLP and GCP are statutory requirements. In contrast, adherence to this GCLP is not a statutory requirement and is not assessed during regulatory GCP laboratory inspections performed by organisations like the MHRA.

## Conclusion

There has been an increased emphasis on the importance of research to the NHS. Pathology’s part in healthcare and research is set to become even more important with diagnostics being central to the rise in genomic, personalised and stratified medicine. It is important that laboratories can demonstrate they have oversight of the research activity undertaken in their department. Quality management, with clear and recognisable processes, record keeping (and therefore traceability) and good communication all support compliance with regulation and guidance. The regulation and guidance around research continues to evolve, reflecting developments in healthcare. It is important that pathologists remain informed about the regulatory requirements, standards and guidance and maintain their professionalism towards the research activities they are involved with. This paper is an overview to highlight some of the regulations and policies around research rather than a comprehensive review. Digital and molecular pathology are areas of research shaping future clinical practice. Through such continued leadership in research, pathology is and will remain a central scientific discipline in clinical practice.

## Data availability

No data are associated with this article.
