# Supplementary material for: Pathology and regulation for research in the UK: an overview
Source: F1000Res. 2019 Sep 19;8:975. Originally published 2019 Jun 27. [Version 2] doi: 10.12688/f1000research.19732.2 (PMC6694446; doi:10.12688/f1000research.19732.2)
Supplement: Peer review history

# Peer review: Reviewer response for version 1

**DOI:** 10.5256/f1000research.21641.r52321

**Published:** 2019-08-28

**Authors:** Tim Helliwell

**Affiliations:** 1 Department of Molecular and Clinical Cancer Medicine, University of Liverpool, Liverpool, UK; 2 Department of Cellular Pathology, Royal Liverpool University Hospital, Liverpool, UK

This article summarises the main regulatory requirements for the successful involvement of pathologists in research. It will be particularly valuable for diagnostic cellular pathologists who are asked to support clinical trials on an occasional basis, as well as for career researchers with a background in pathology.

 Pathologists should be aware that the success or failure of a research project may depend on the quality of material that is analysed; as experts in the handling of tissues and fluids, pathologists are key members of the research team. For human tissues, the conditions of transport and storage (frozen, paraffin wax blocks) may influence the results of research investigations just as much as investigations for clinical practice.

 Specific comments:

1. Figure 1 clearly indicates the potential extent of involvement by pathologists. The accompanying text does not pick up on the 'right hand' green box in the figure which emphasises the opportunity for intellectual input into discussing the results. The scientific understanding provided by pathologists can offer caution in interpreting results, if appropriate, where biological factors e.g. tumour sampling variability, may influence conclusions. A clinician scientists, pathologists are also in a position to discuss the possible clinical impact of the results.
2. It might be useful if the authors could state how much of the article is aimed at cellular pathologists and which areas might also be relevant to the other pathology specialities.
3. In the section on accreditation, ISO 15189 is more correctly cited as ISO 15189:2012.
4. Accreditation to the ISO standard implies, as stated in the article, the implementation of an appropriate quality management system. This will include (in the appropriate context) seeking evidence on how a laboratory ensures that research samples are appropriately handled and stored. Factors influencing the uncertainty of measurement are familiar to laboratories, and might impact on the assurance that research sponsors require that samples are of optimal quality for research as well as diagnosis.

I confirm that I have read this submission and believe that I have an appropriate level of expertise to confirm that it is of an acceptable scientific standard.

# Peer review: Reviewer response for version 1

**DOI:** 10.5256/f1000research.21641.r50491

**Published:** 2019-08-12

**Authors:** Bridget Wilkins

**Affiliations:** 1 St Thomas' Hospital London and Royal Hampshire Country Hospital, Winchester, UK

This is a valuable brief summary of the regulatory landscape in the UK within which research use of pathology-related biosamples sits. It highlights the core contributions of the national bodies that regulate clinical trials, and the requirements of laboratory accreditation in the UK that underpin quality assurance of pathology samples. The cited sources will provide a useful and regularly updated source of information regarding MHRA, HRA, HTA and UKAS/ISO 15189. 

 I think that it would be helpful to extend the focus more explicitly beyond the field of clinical trials, to provide confidence for pathologists working in diagnostic settings who wish to undertake research work unrelated to clinical trials. To this end, a little more discussion about how NHS pathology laboratories can achieve HTA licensing for research storage of 'surplus' tissue, the topic of generic/specific consent, and HRA processes for achieving ethical review of research (particularly the processes for research deemed to raise minimal ethical concerns) would all be valuable.

 Legitimate access to a useful dataset of clinical information to accompany tissue samples is often a perceived barrier to pathology-based research, which could usefully be 'myth busted' in an article such as this (HRA and GDPR considerations, in particular).

 In my opinion, the topics I've touched on above are all inextricably linked to the regulation of tissue-based research and to the encouragement of pathologists to engage in well-regulated research - the 'holy grail' of the NCRI's CM-Path initiative.

I confirm that I have read this submission and believe that I have an appropriate level of expertise to confirm that it is of an acceptable scientific standard.

# Response

**Published:** 2019-08-12

**Authors:** Owen Driskell

**Affiliations:** NIHR CRN West Midlands, UK

We thank the reviewer and agree with their comments.

The constraints in the scope for this individual article did not permit an exhaustive exploration of the wider details specifically facing NHS laboratories hosting diagnostic archives. We agree wholeheartedly they represent a great resource for potential research that would benefit NHS services and ultimately patients and the care they receive. Further clarity in education and ‘myth-busting’ will benefit the cause of breaking down the perceived barriers to pathology-based research.

# Peer review: Reviewer response for version 1

**DOI:** 10.5256/f1000research.21641.r50493

**Published:** 2019-07-11

**Authors:** Mark Sobel

**Affiliations:** 1 American Society for Investigative Pathology (ASIP), Rockville, MD, USA

The Opinion Article by Owen Driskell and colleagues cites and briefly explains the relevant regulations in the UK concerning research conducted in pathology departments, with an emphasis on clinical trials. Editorially, and consistent with the principles of the UK policy framework for health and social care research (reference 1 - https://www.hra.nhs.uk/planning-and-improving-research/policies-standards-legislation/uk-policy-framework-health-social-care-research/) the authors state that the “requirements of the regulatory environment should not be seen as a barrier to research and should not significantly impact on the work of the laboratory once established and integrated into practice.”

 The article is organized, generally well written and succinct, and provides links (in the references) to the appropriate statutes and regulations.

 This reviewer recommends (in the section “Human Tissue Act 2004 and the Human Tissue Authority”) that the authors clarify whether the Human Tissue Act covers analytes stored in pathology departments such as DNA, RNA, and protein that have been isolated from human tissues.  The Human Tissue Act does not consider acellular samples to be “relevant material;” however the Act clearly covers the use of DNA in research.

Editing suggestions for consideration by the authors:

- Abstract (sentences 2-3): “As the custodians of patient samples, pathology departments have a duty to ensure compliance with the relevant regulations, standards and guidelines to ensure the ethical and effective use for their intended investigational analysis, including when patients are participating in a research study.”
- Paragraph 2 (sentences 1-2): “In addition to research initiated by pathologists, a large amount of research is led by researchers from outside pathology requiring services delivered by pathology departments.”
- Paragraph 2 (sentence 4): “Data generated from pathology departments support research in a wide variety of ways.” (“Data” is plural.)
- Paragraph 13 (beginning “The primary focus of inspection by the MHRA…”) the second and third sentences could be misinterpreted. I suggest rewording as follows:  “Although routine sample analyses for safety testing within clinical chemistry and haemotology laboratories are not the central focus of this inspection programme, the legislation does not differentiate between the purpose of different types of sample analysis; this is a pragmatic approach by the MHRA Inspectorate and potentially any aspects may be reviewed during an inspection.”
- Paragraph 17 (sentence 2): All the existing professional standards and regulations still apply and those that relate specifically to research must also be applied.”

I confirm that I have read this submission and believe that I have an appropriate level of expertise to confirm that it is of an acceptable scientific standard.

# Response

**Published:** 2019-08-12

**Authors:** Owen Driskell

**Affiliations:** NIHR CRN West Midlands, UK

Thank you for the reviewers helpful comments.

 In response to the question of DNA.

 DNA itself - as opposed to the cellular material from which it originates - is not considered to be relevant material under the HT Act. Its storage is therefore not subject to HTA licensing.

 However, the reviewer is correct. The Act does state to anyone holding cellular material without the qualifying consent of the person/s concerned, intending to analyse the DNA and use the results, may be breaking the law. It is an offence to analyse DNA without qualifying consent unless it is for an excepted purpose.

 Having human tissue, including hair, nail, and gametes (i.e. cells connected with sexual reproduction), with the intention of its DNA being analysed without the consent of the person from whom the tissue came or of those close to them if they have died is DNA Theft (Medical diagnosis and treatment, criminal investigations, etc., are excluded).

 Appropriate informed consent is at the heart of Human Tissue Regulation and at the heart of Research Regulation. Pathology professionals must understand their role in research, and the systems in place by which informed consent is achieved, to give confidence to their compliance with this regulation. 

 RNA is not covered by the Human Tissue Act 2004 but the guidance produced by HTA also applies to RNA analysis where it is to be used to provide information about DNA.
